# Assessment of analytical performance of glucose meter in pediatric age group at tertiary care referral hospital

**DOI:** 10.1186/s40200-017-0318-2

**Published:** 2017-09-13

**Authors:** Mohit Vijay Rojekar, Vandana Kumawat, Jayesh Panot, Surekha Khedkar, Arati Adhe-Rojekar

**Affiliations:** 1 Rajiv Gandhi Medical College, Thane, India; 2 PD Hinduja National Hospital and Research Center, Mumbai, India

**Keywords:** Glucometer, Parke error grid, Surveillance error grid

## Abstract

**Background:**

Glucometers are the excellent tools for self-monitoring of blood glucose (SMBG). They are important especially in the circumstances where continuous monitoring is mandatory and at decision making levels. Tight glycemic control protocols are important for preventing the ill effects of fluctuating glucose levels. This increases the use of glucometers in various healthcare settings. As technology advances, glucometers are getting better in terms of quality of results. But still some lacunae are there.

**Methods:**

Present study was conducted in the tertiary care referral hospital. One hundred twenty five patients were recruited from pediatric wards. Bland-Altman plot, Parke error grid and Surveillance error grid analysis were used for comparing results of glucose meter with that of standard laboratory method.

**Results:**

It is found that there is significant difference between the results by two methods. Though minimal but glucose meter results deviate from the results of standard lab method. This will affect the overall patient care especially in emergency conditions.

**Conclusions:**

This study is the first of its kind as no similar studies have been reported in the pediatric population. For effective use of glucose meter it should give as accurate as possible estimate of actual glucose levels. Results should not only be accurate but also precise without which critical errors may be possible. We recommend that for any glucose meter there should be regular maintenance as well as calibration is to be done. So that agreement with reference laboratory method is maintained and effective medical decisions are made.

## Background

Glucometers are the excellent tools for self-monitoring of blood glucose (SMBG), especially in the circumstances where continuous monitoring is mandatory and in decision making. Glucometer is very useful at the time of emergencies when immediate glucose levels are needed for assessment. SMBG is especially important for patients treated with insulin to monitor glucose levels and prevent asymptomatic hypoglycemia and hyperglycemia. Glucose control is very crucial in managing diabetes mellitus in pediatric, surgical and medical Intensive care units (ICU) where continuous monitoring of blood glucose is required [1, 2] as patients need to achieve and maintain glycemic control, prevent and detect hypoglycemia and if required adjust changes in lifestyle [1]. Tight glycemic control protocols are important for preventing the ill effects of fluctuating glucose levels. This increases the use of glucometers in various healthcare settings.

Glucometers in which glucose strips are biosensors in that they all use an enzyme as the recognition agent; glucose oxidase-peroxidase (GOD-POD), hexokinase (HK), or glucose dehydrogenase (GDH) with photometric (reflectance) or, more commonly, electrochemical detection may be used [3].

Usually the glucometers replaced the routine lab analysis in settings where quick result is necessary. As technology advances, glucometers are getting better in terms of quality of results but still some lacunae are there in the form of variations. Various brands of glucometers are available in the market. There is significant difference among these devices as variation in accuracy and precision. There are guidelines issued by International Standardization Organization (ISO) and American Diabetes Association (ADA) for the use of glucometers. The ISO 15197:2003 standard requires that 95% of the values be accurate within ± 15 mg/dL for glucose values < 75 mg/dL and within ± 20% for glucose values ≥ 75 mg/dL. These were updated in 2013 (ISO 15197:2013) to require 95% of values to be accurate within ±15 mg/dL for glucose values < 100 mg/dL and within ± 15% for glucose values ≥ 100 mg/dL [4, 5]. ADA limits the accuracy to variation of ± 5% irrespective of results [1]. Efforts were put to determine accuracy and precision of the glucometers using various statistical methods like correlation and regression. [6] Mere trend of the readings is not important many a times where actual correct values are needed. This is not properly answered by correlation or regression tools of statistics. This is why error grid analysis was introduced in mid 80s. Specific risk is assigned to possible errors in blood glucose measurement. In this actual and estimated blood glucose are associated with five risk levels [7, 8]. Therefore we have decided to look into the quality of the results of glucometer in our tertiary care referral hospital. Here we checked whether results by glucose meter are in agreement with the results of laboratory analysis.

## Methods

Present study was conducted in the Tertiary Care Referral Municipal hospital in Thane district of Maharashtra, India. We have recruited 125 patients from pediatric ward, NICU, PICU in this study. Selection was random to avoid any selection bias. All the patients were enrolled after obtaining the written informed consent from either their parents or caretakers as per Helsinki declaration. The random whole blood venous sample was collected in sodium fluoride bulb. Plasma samples from these patients were analyzed for glucose concentration by the glucometers and by GOD-POD method in the biochemistry laboratory simultaneously. This is to provide same sample for both methods. This will help to eliminate the bias of inherent difference for glucose estimation between capillary blood and venous blood. Efforts were made to cover all ranges of plasma glucose in the samples that were collected.

Bland-Altman method was used to plot the bias (the difference between the glucometer reading and the plasma glucose concentration measured by the reference method) against the corresponding glucose mean [9]. Error grid was plotted according to Parke’s error grid analysis and Surveillance error grid protocols for the assessment of clinical utility of test [6]. Paired t-test was used to compare the difference of bias between the glucometer reading and the reference method. *P* value was taken as < 0.05 for the results to be statistically significant. Statistical analysis was performed using software SPSS 20.0. ISO 15197:2003 and 2013 guidelines were used to assess the acceptability of the results [10, 11]. Mean absolute relative error (MARE) was also calculated as per ISO criteria 2013 [12].

## Results

As shown in Table 1 there is statistically significant difference between the means of glucometer and GOD-POD results.

**Table 1:** Blood glucose level by reference method and glucometer

	GOD-POD Method	Glucometer	*p* Value
Blood Glucose Levels (mean ± SD)	87.58 ± 20.31	96.58 ± 19.63	< 0.001

In Parke Error Grid Analysis (P-EGA), x-axis is defined as reference method (GOD-POD) measured blood glucose and the y axis as the value obtained by the glucometer. In above diagram P-EGA shows distribution of results of glucose estimation by glucometer versus that by GOD-POD method. The dotted line shows exact agreement between the two i. e. glucometer and GOD-POD method.

The error grid is divided into five zones of clinical accuracy; including the appropriateness of the laboratory result. The risk categories, in order of increasing severity, were defined as follows: A: no effect on clinical action; B: altered clinical action or little or no effect on clinical outcome; C: altered clinical action—likely to affect clinical outcome; D: altered clinical action—could have significant medical risk and lead to dangerous medical consequences [7].

**Fig. 1:**
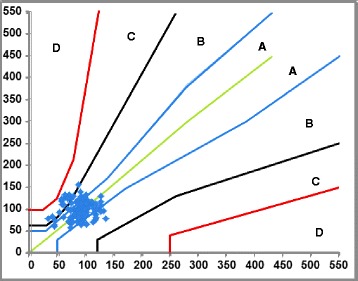
Parke’s error grid analysis

Twenty six results i.e. 20.8% in Fig. 1 are in zone ‘B’. It means there is altered clinical action but little or no effect on clinical outcome. Fourteen results i.e. 11.2% are found to lodge into zone ‘C’. Here there is altered clinical action and likely to affect clinical outcome. This means errors in this zone lead to unnecessary treatment when actually no corrections are needed. In short this is overcorrections of normal glucose levels.

**Fig. 2:**
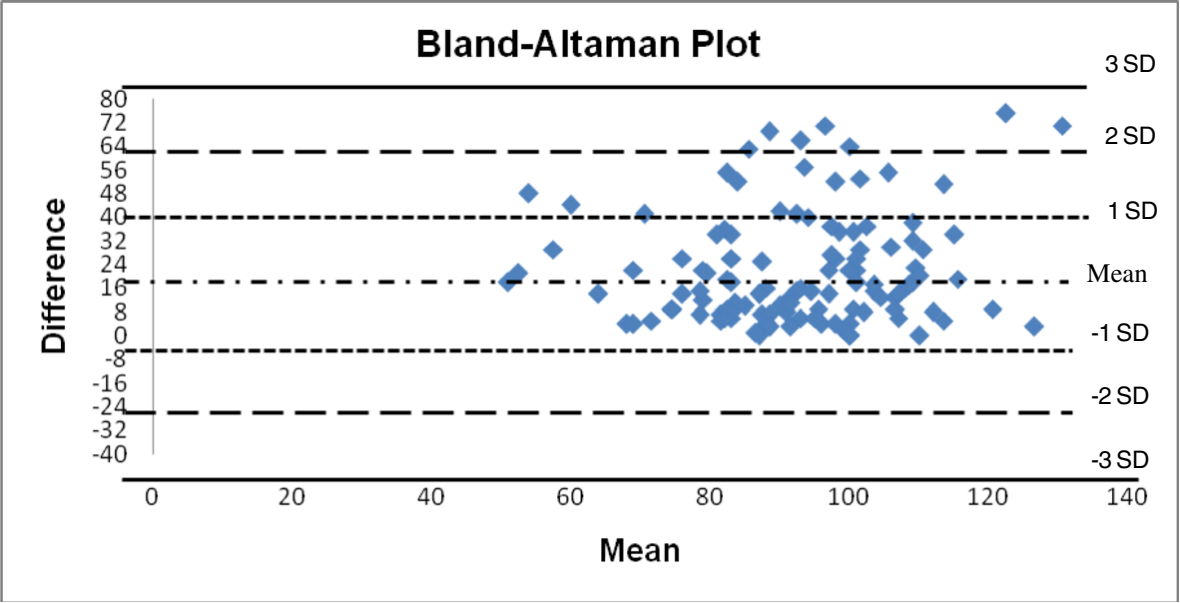
Bland-Altman plot

Figure 2 shows Bland-Altman (BA) plot which is usually understood as a plot of the differences against the average results of the methods [13, 14]. Thus the BA plot in this version provides information on the relation between differences and concentration which is useful in evaluating whether problems exist at certain ranges caused by nonlinearity of one of the methods [15]. In Fig. 2, BA plot shows a tendency toward increasing scatter with increasing concentration, which is a reflection of increasing random error with concentration. Most of results are within **±** 1 SD while very few are between **+** 2 SD and **+** 3 SD.

**Fig. 3:**
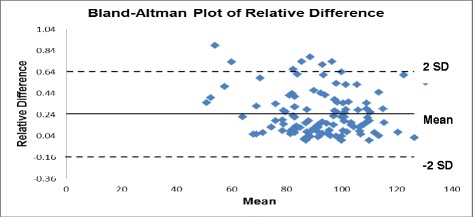
Bland-Altman plot of relative difference

In Fig. 3 the BA plot of relative difference is shown. Here plotting the relative difference prevents very large differences in the high concentration range from dominating the analysis. Similar to BA plot, plot of relative difference also shows distribution of results between **±**2 SD while few are beyond **+** 2 SD.

**Fig. 4:**
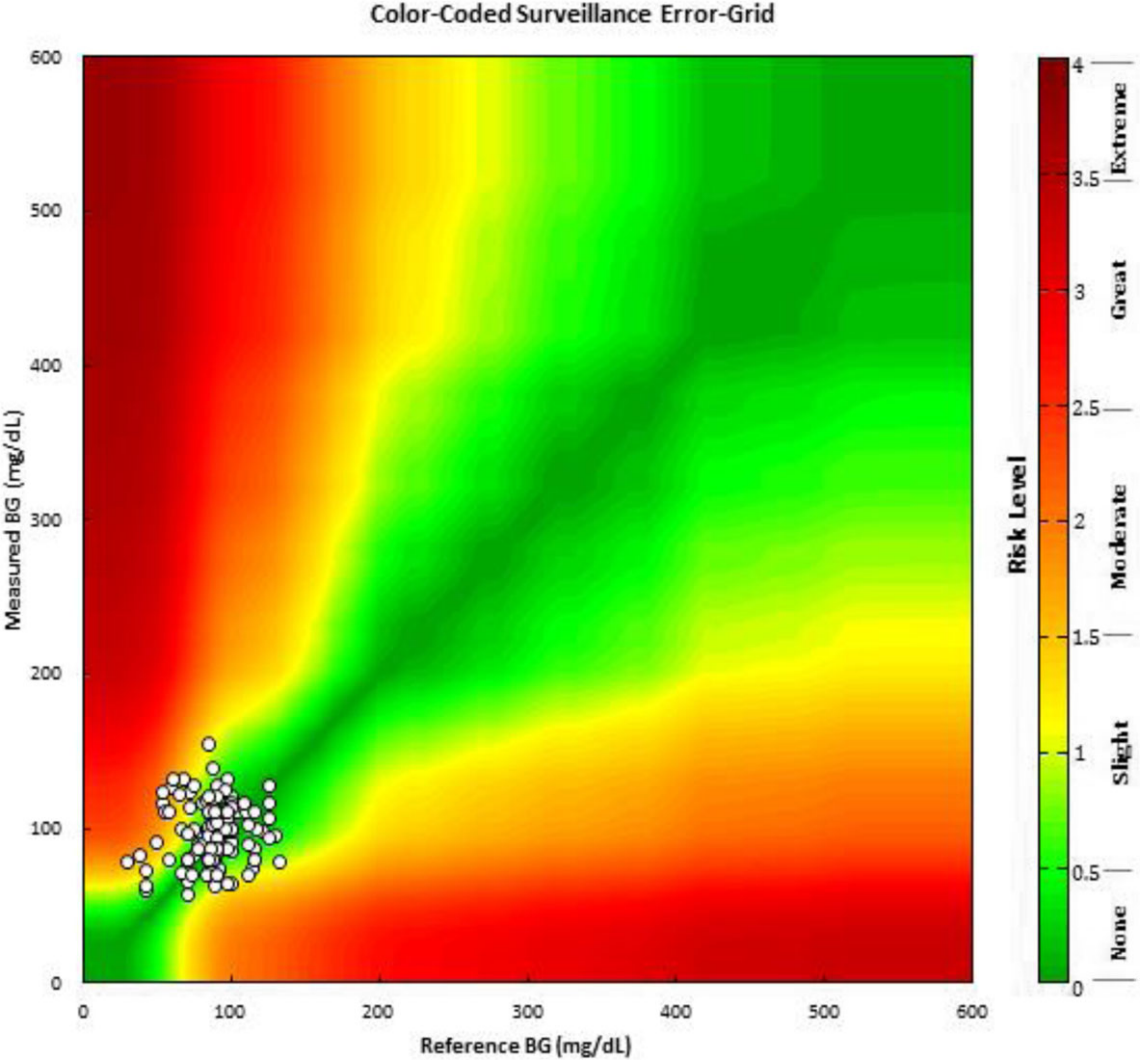
Surveillance Error Grid (SEG)

A classic error grid maps risk zones on a two-dimensional graph, whereas the Surveillance Error Grid (SEG) as shown in Fig. 4, is a three-dimensional graph plotted on two dimensions, with the third dimension being color. It uses risk scores to individually account for each data point which will produce a more granular analysis of clinical performance of BG monitor. With the SEG, a difference among results could be noted because of the continuity and the greater granularity of the analysis. Using the continuous scoring feature of this grid, it is also possible to define any risk score that one wishes to specify to determine the percentage of data points falling above and below this risk level [16]. In SEG, 5-9 zones could be made depending upon risk severity.

Table 2 shows distribution of the results in Surveillance Error Grid analysis. Out of 125, 77 results have no risk of hypo or hyperglycemia while 23 results are having slight lower risk and 14 having slight higher risk. Next group of moderate low risk have 10 results while group of moderately high risk have only one result.

**Table 2:**
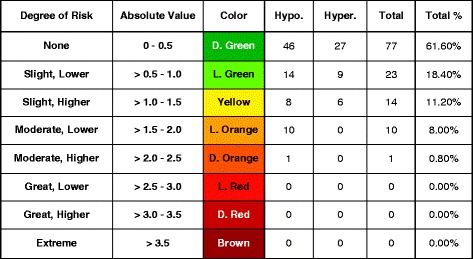
Surveillance error grid

**Fig. 5:**
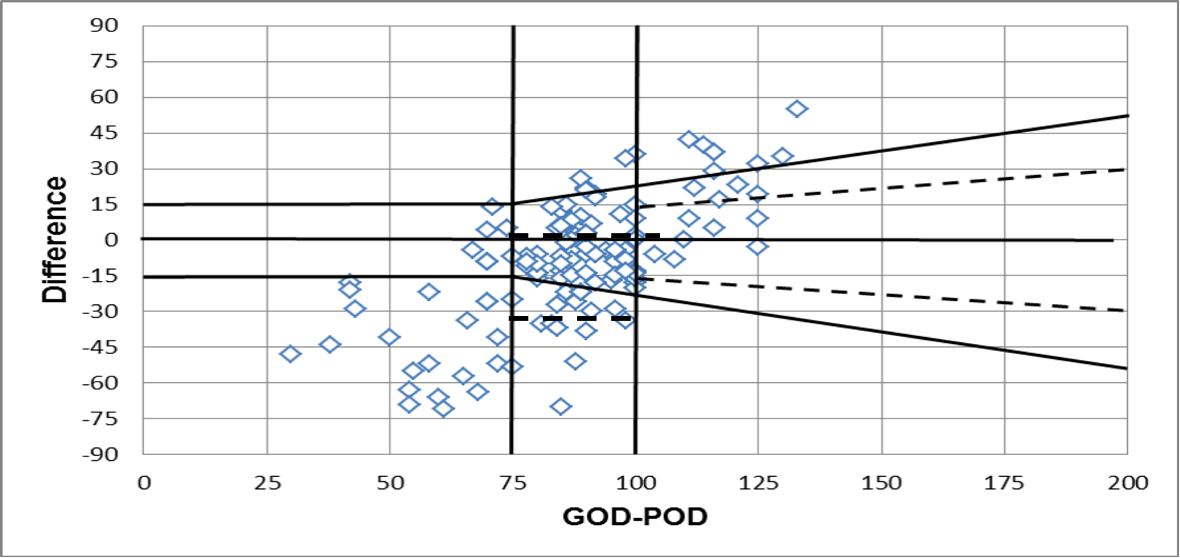
ISO 15197:2003 and ISO 15197:2013

In Fig. 5 dotted lines represent distribution of the results as per ISO 15197:2013 while dark line represents according to ISO 15197:2003.

**Fig. 6:**
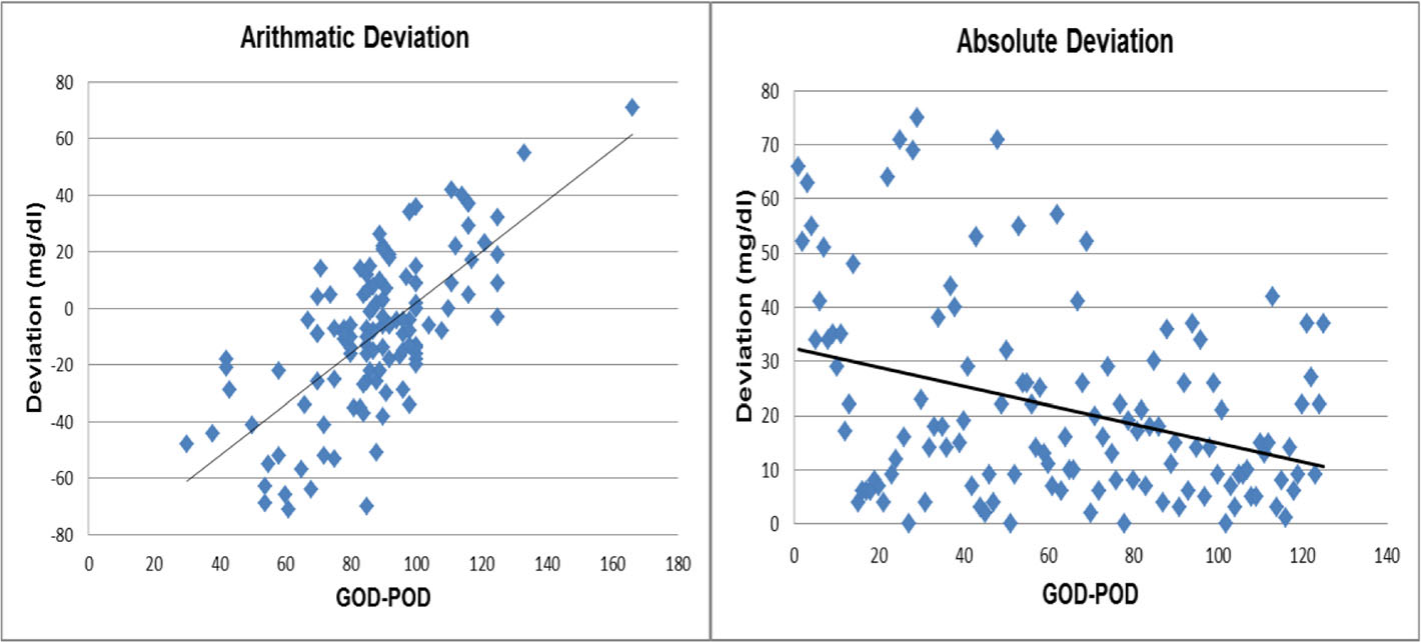
Arithmatic deviation & absolute deviation

Figure 6 shows the arithmatic and absolute deviation respectively of glucometer results from refernce method. Arithmatic deviation shows the difference goes on increasing as ocncentration increases. While absolute deviation decreases as concentration goes on increasing.

## Discussion

According to SEG, 80% of our results are not problematic. 60% of results have no risk while 20% have very negligible risk. This is far better than many other studies. Many regulatory authorities have established standards for the testing accuracy of glucose monitors. These criteria differ significantly, ranging from high tolerance of up to 20% from the reference value as per Clinical and Laboratory Standards Institute (CLSI) guidelines to a deviation of only 5% as per American Diabetes Association (ADA) guidelines.

Glucose meters are the standard practice for monitoring of blood glucose concentration and management in hypoglycemia, hyperglycemia and diabetic patients. Using a single drop of blood, glucose meters provide rapid test results for immediate medical decisions and prompt initiation of therapy. They are widely available for patient for self-monitoring, in various hospital setups, NICUs, PICUs and ICUs where prompt diagnosis and management of hypoglycemia, hyperglycemia and diabetes is essential. Many glucose meters were first introduced to the market for patient self-testing, and later the same methodology was used in glucometers intended for professional use in a hospital setting. The analytical performance in home settings for patient self-testing may not meet the same needs for hospitalized patients, given the physiological differences between these patient populations (eg, hematocrit ranges, oxygenation, perfusion, drugs and metabolites). Glucose meters have been promoted for management of inpatient glycemic control in various hospiatal settings in consensus statements from the American Association of Clinical Endocrinologists and the American Diabetes Association [1].

Glucose meters are very convenient and fast in diagnosis but have some limitations as many factors contribute to variability and accuracy of glucose meter results. These include operator technique, environmental exposure, and patient physiologic and medication effects [11]. Extremes of hematocrit can variably affect glucose readings depending on the methodology and initial concentration of glucose [17, 18]. Naturally occurring substances like triglycerides (TG) and paraproteins can give falls low value while high uric acid gives falls high value as it gets oxidized in glucose meters [19]. Similarly oxygen therapy can affect meters with glucose oxidase reagents [11]. Drugs like high doses of ascorbic acid can interfere when used burn patients [20]. Also Maltose and dopamine have found to interfere with glucose dehydrogenase based glucose meters [11, 21]. Other sugars, like galactose, can also be sensed as glucose by some meter reagent enzymes, falsely elevating test results. Exposure of test strips to heat, cold, light, humidity, altitude, and other environmental factors can variably affect glucose meter results [11]. These are the few examples which affect the readings of glucometers.

Accuracy is difficult to establish in glucometers as it uses whole blood and glucose in whole blood is unstable. Transportation delay can be one of the causes of difference in results of two methods. Glycolysis by RBCs consumes and reduces the glucose levels at the rate of 5-7% per hour [22]. Fluoride is used to prevent glycolysis but its action starts after 1-2 h as this much time is required for crossing RBC membrane.

One more alarming issue is lack of regular calibration of glucose meters. This could be taken care by sticking to user guidelines provided by manufacturer [23]. It is reported that nearly 91-97% of all inaccuracies are due to operator like faulty meters, inappropriate cleaning of test site and sample related issues like bubbles or clots in sample and inadequate application of the sample [24, 25].

Lacunae of our study are not testing the sample withdrawn for lab analysis simultaneously by glucose meter. This is because there is inherent difference between the capillary and venous blood glucose levels. That would have been more informative if done. Also we have not calibrated the glucose meters before use because we want to check the result in actual settings of glucose meter use.

## Conclusion

This study is the first of its kind as no similar studies have been reported in the pediatric population. As glucose meters are prompt in estimation of blood glucose levels, they are the best POCT tools available for management of hypoglycemia, hyperglycemia and diabetes mellitus. Glucose meters can be used in diverse group of patients. Even a person with no medical knowledge can use it effectively.

For effective use of glucose meter it should give as accurate as possible estimate of actual glucose levels. Results should not only be accurate but also precise without which critical errors may be possible. Accuracy and precision definitely adds up to effective medical decisions. We therefore recommend that for any glucose meter there should be regular maintenance as well as calibration is to be done. So that agreement with reference laboratory method is maintained and effective medical decisions are made.

## Abbreviations

ADA: American Diabetes Association
GDH: Glucose dehydrogenase
GOD-POD: Glucose oxidase peroxidase
HK: Hexokinase
ICU: Intensive care unit
ISO: International Standardization Organization
SEG: Surveillance error grid
SMBG: Self-monitoring of blood glucose
